# Blanching Effect on the Quality and Shelf-Life Characteristics of Fresh Cowpea Grains [*Vigna unguiculata* (L.) Walp.]

**DOI:** 10.3390/foods11091295

**Published:** 2022-04-29

**Authors:** Romayana Medeiros de Oliveira Tavares, Cristiane Fernandes de Assis, Patrícia de Oliveira Lima, Paulo Douglas Santos de Lima, Roberto Rodrigues Cunha Lima, Karla Suzanne Florentino da Silva Chaves Damasceno

**Affiliations:** 1 Health Sciences Center, Nutrition Postgraduate Program, Department of Nutrition, Federal University of Rio Grande do Norte, Av. Senador Salgado Filho, s/n, Lagoa Nova, Natal 59078-900, Brazil; romayana.tavares@ufrn.br; 2 Health Sciences Center, Nutrition Postgraduate Program, Department of Pharmacy, Federal University of Rio Grande do Norte, Av. Senador Salgado Filho, s/n, Lagoa Nova, Natal 59078-900, Brazil; cristianeassis@hotmail.com; 3 Postgraduate Program in Animal Science, Department of Animal Sciences, Federal Rural University of the Semi-Arid. Rua Francisco Mota, 572, Pres. Costa e Silva, Mossoró 59625-900, Brazil; pattlima@ufersa.edu.br; 4 Department of Theoretical and Experimental Physics, Federal University of Rio Grande do Norte, Av. Senador Salgado Filho, s/n, Lagoa Nova, Natal 59078-900, Brazil; paulo.douglas.lima@fisica.ufrn.br; 5 Federal Institute of Education Science and Technology of Rio Grande do Norte, Rua Brusque, 2926, Potengi, Natal 59112-490, Brazil; roberto.lima@ifrn.edu.br

**Keywords:** heat treatment, quality control, enzymatic activity, legume, shelf life

## Abstract

The high perishability of fresh cowpeas impairs its commercialization. Thus, this study aims to determine the temperature–time binomial for blanching fresh cowpea [*Vigna unguiculata* (L.) Walp] by evaluating the effects of heat treatment and kinetic behavior on the peroxidase (POD) activity and on the physical characteristics (firmness, color, mass gain). A factorial design (3 × 6) with temperature (70, 80, and 90 °C) and time (1, 2, 4, 6, 8, and 10 min) was implemented. Physicochemical, microbiological, and enzymatic (POD) changes, in addition to photographic monitoring, were evaluated throughout the storage period (4.90 °C). With regard to the effects of the independent variables and the first-order kinetic model, it was determined that 70 °C for 4 min of blanching maintained and/or improved the physical characteristics of the raw material. The pH and the acidity of the blanched fresh cowpea changed little during the storage period; the microbiological load and POD activity reduced with blanching and remained stable until the eighth day of storage, and provided an increase of 5 days in shelf-life under refrigeration when compared to non-blanched. Blanching is shown as an alternative for improving fresh cowpeas, favoring an economic increase in production with guaranteed quality and safety attributes.

## 1. Introduction

World food consumption continues to increase, and therefore the concern for healthy and good quality food has been increasing [1]. It is possible to highlight the importance of legumes among the food groups, which play an important role in the world market by adding socioeconomic and nutritional value to the population [2].

The cowpea [*Vigna unguiculata* (L.) Walp] is one important grain legume cultivated worldwide and stands out for its soil adaptability in line with production systems in tropical and dry regions which cover parts of Asia, the South of the United States, the Middle East, and Central and South America [3]. It is an important source of carbohydrates and proteins, standing out for its dietary fiber, vitamin, and mineral content, in addition to having a low amount of lipids [4].

Cowpeas have a limited shelf-life in their green ripening stage, and only retain their physical–chemical and quality properties (color and texture) for a maximum of 3 days when stored under refrigeration. The main causes of degradation are high humidity and the enzymatic activity of the fresh grains, which makes the raw material perishable, thus generating difficulty in marketing, and in turn predisposes them to waste.

According to the United Nations Environment Program’s global estimate, about 931 million tons of food were wasted in 2019 [5]. As a result, the quest to reduce food waste will be paramount to achieving sustainable development goals. Waste is related to consumer education, as well as to sustainable production and food distribution [6].

Vegetables are affected by several processes after harvest, and quality attributes such as texture, color, flavor, nutrients, and bioactive compounds are affected by the action of intracellular enzymes such as peroxidase (POD) and polyphenoloxidase (PPO) [7,8].

Blanching has been widely used in different foods in order to optimize the preservation processes and contribute to enzymatic inactivation and microbiological control [9]. The technique acts in enzymatic reduction or inactivation [10], in reducing the microbial load [11], and the color is highlighted and the texture is less altered with the correct use of time and temperature, thus maintaining the vegetable/legume quality properties [12].

Zhang et al. [13] reports that fresh green legumes require blanching to reduce quality deterioration before storage due to high humidity and the activity of endogenous enzymes. The authors investigated the effects of radiofrequency blanching on the relative activities of lipoxygenase (LOX) and POD of green peas, and found a significant decrease in the relative activity of the enzymes with bleaching at 85 °C. However, the color, texture and electrolyte leakage of peas changed significantly with increasing temperature (60–85 °C).

Due to the thermostability of the peroxidase enzyme, it has been used as an indicator of the blanching [14]. However, inactivation or a marked decrease in peroxidase activity can compromise the quality properties of vegetables such as texture and color [15].

According to Gonçalves et al. [16], it is essential to develop kinetic studies and investigate the effects of temperature and time on the extent of changes in physical and quality properties, as well as the enzymatic activity of raw material in order to optimize the blanching treatment and have an efficient approach to the technique.

Therefore, defining methodological procedures and studying the effects and kinetics of blanching fresh cowpeas is important to ensuring scientific support for applying this technique, with respect to the physical–chemical standards of the legume, as well as the quality attributes for production, transport, marketing, and consumption.

In this perspective, improvement in the quality of fresh cowpea is an alternative, not only in commercial terms, but also to optimize procedures which aim at the quality of food for self-consumption, enabling obtaining food with organoleptic characteristics of the product in natura, microbiologically safe, and at the same time, favoring conservation and extension of the product’s shelf-life.

There are a few studies in the literature on fresh cowpea and even fewer which specifically use pretreatments to improve food stability for consumption and commercialization. In view of this, the present study aims to investigate the effects of blanching on physical characteristics (firmness, color and mass gain) and on the peroxidase enzymatic activity in fresh cowpeas; to study the kinetic behavior of these properties; and to evaluate the shelf-life of fresh and blanched fresh cowpeas stored under refrigeration.

## 2. Materials and Methods

The study was conducted in two stages: Phase 1—Studying the effects of blanching on fresh cowpeas and a kinetic evaluation of the variables enzyme activity, firmness, and color. Phase 2—Determining the shelf-life of the product stored under refrigeration.

### 2.1. Vegetable Material

The raw material was in natura cowpea grains (*Vigna unguiculata* L. Walp.), provided by a producer in the municipality of Boa Saúde, Rio Grande do Norte, Brazil. This study was registered in the National System for the Management of Genetic Heritage and Associated Traditional Knowledge, under number AF4C703. A total of 45 kg of fresh cowpeas were used in the study. The grains were received less than 15 h after the harvesting, threshing, and bagging process, and then kept at room temperature (24 °C) during the selection process according to the following quality standards: no physical damage, cracks, or perforations, green ripeness, and uniform size and color.

### 2.2. Methods Phase 1 Studying the Effects of Blanching on Fresh Cowpeas and a Kinetic Evaluation of the Variables Enzyme Activity, Firmness and Color

#### 2.2.1. Blanching Process

First, a 3 × 6 factorial design was performed for the fresh cowpeas blanching tests (two factors, temperature at three levels (70, 80, and 90 °C) and time at six levels (1, 2, 4, 6, 8, 10 min), generating 18 tests which were performed in random order and in duplicate.

The fresh cowpeas were randomly separated into samples of 200 g. The samples were then subjected to heat treatment in a thermostatic water bath (Luca-150/10D, Lucadema^®^, São Paulo, Brazil), being immersed in hot water distilled in proportion between sample weight and water volume of 100 g/L, which were subsequently drained in a stainless steel sieve and immediately cooled in an ice bath until reaching a temperature of 10 °C. They were packaged and stored under refrigeration [4.90 °C (0.32)] until analysis. The samples were kept frozen (−18 °C) for the enzymatic activity analysis. The samples were analyzed with a maximum of 24 h of storage.

#### 2.2.2. Extraction and Peroxidase Analysis

Extraction and peroxidase analyses were performed in the first and second phases of the study. The crude enzymatic extract was obtained and the peroxidase (POD) analysis was conducted according to what was proposed by Bonnely et al. [17] and Campos and Silveira [18], with modifications. The samples (2 g) of fresh cowpeas were homogenized (1 min) in a mortar in an ice bath with 1.6 g of polyvinylpyrrolidone (PVP K30) and 30 mL of 0.05 mol. L^−1^ phosphate buffer solution at pH 7.0. The obtained dispersion was centrifuged at 4500× *g* for 20 min under refrigeration (4 °C) (Centrifuge 5804R, Eppendorf^®^, Hamburg, Germany) and subsequently filtered. The supernatant (crude extract) was used to determine the POD activity.

Peroxidase activity was analyzed from the reaction mixture of 2500 μL of phosphate-citrate buffer solution (0.05 mol·L^−1^, pH 5.0), 100 μL of crude extract, 250 μL of guaiacol (0.5%), and 250 μL of H_2_O_2_ (3%). The mixture was shaken and incubated in a water bath (Q334M-28, Quimis^®^, São Paulo, Brazil) at 30 °C for 15 min, followed by cooling in an ice bath, adding 250 μL of sodium metabisulfite solution (2%) and resting for 10 min. The enzymatic activity was determined by reading on a spectrophotometer (Digital Sp-220, Bioespectro^®^, Paraná, Brazil) at 450 nm in triplicate. The crude extract was replaced with distilled water for a blank. One unit of enzyme activity is defined as the amount of crude extract which showed an increase in absorbance of 0.001 unit per minute.

The enzyme activity was expressed in an enzyme unit (*U/g*) (Equation (1)).

(1) $$U/g=\frac{A}{\epsilon }\times \frac{1}{Ve}\times DF\times \frac{1}{t}\times 1000\times \frac{Et(\mathrm{mL})}{M(g)}$$

In which: *U*/*g* = unit of activity per g of fresh cowpeas; *A* = absorbance; *ϵ* = molar absorptivity of tetraguaiacol (26,600 mol^−1^ cm^−1^); *Ve* = volume of the enzyme solution used in the assay (mL); *DF* = dilution factor (dilution of crude enzyme extract); *t* = reaction time in minutes; *Et* (mL) = total yield of the crude extract in mL; and *M* (g) = mass of grains used for the crude extract.

The relative POD activity was calculated according to Feng et al. [11] (Equation (2)).

(2) $$\mathrm{Relative}\;\mathrm{Enzymatic}\;\mathrm{Activity}\;(\mathrm{REA}\%)=\frac{At}{Ao}\times 100\%$$

In which: *At* is the POD activity of the blanched sample, and *Ao* is the enzyme activity of the in natura sample.

#### 2.2.3. Instrumental Firmness Analysis

The instrumental firmness analysis was performed with a texturometer (TA, XT Express, Stable Micro Systems^®^, Surrey, UK) equipped with a 2 mm diameter cylindrical probe. The equipment was previously calibrated with a 2 kg load cell. For puncture tests, the fresh cowpeas were drilled on a stainless steel platform at a rate of 2 mm/s with a trigger force of 0.049 N. The analysis was performed according to Resende, Corrêa, Ribeiro, and Neto [19], with modifications. The fresh cowpeas were drilled in three different positions (Figure S1). We used 5 grains for each axis, totaling 15 grains per sample. The data are expressed as maximum drilling force in Newton (N).

#### 2.2.4. Mass Gain

The fresh cowpeas were weighed before blanching and immediately after cooling. The water was drained before weighing. The results are presented as a percentage of mass gain (Equation (3)).

(3) $$\mathrm{Mass}\;\mathrm{gain}\;\left(\%\right)=\left[\frac{M_{1}{-M}_{0}}{M_{0}}\right]\times 100$$

In which: *M*_0_ is the initial mass of the untreated sample (in natura cowpeas) and *M*_1_ represents the mass of the treated samples (blanched fresh cowpeas).

#### 2.2.5. Instrumental Color Analysis

The color of the fresh cowpea grains was measured before (in natura cowpea) and immediately after blanching using a portable spectrophotometer (CM-700d, Konica Minolta^®^, Tokyo, Japan). The color was expressed according to the codification of the International Commission on Illumination (CIE) by L* (Luminosity), a* (red-green) and b* (yellow-blue). The chroma index was defined to assess saturation (Equation (4)). The spectrophotometer was adjusted with a white calibration standard (CM-A177, Konica Minolta^®^, Tokyo, Japan).

(4) $$\mathrm{Chroma}=\sqrt{a^{*2}{+b}^{*2}}$$

#### 2.2.6. Mathematical Standards for Kinetic Analysis

The experimental data of the enzymatic activity and the variations in firmness and color were applied to different kinetic models to verify the fit of the results to the models of zero order (Equation (5)), first order (Equation (6)), or fractional first order (Equation (7)).

(5) $${C(t)=C}_{0}-kt$$

(6) $$\frac{C}{C_{0}}{=e}^{-kt}$$

(7) $${C(t)=C}_{eq}+\left(C_{0}{-C}_{eq}\right)e^{-kt}$$

In which: *C* corresponds to the evaluated quality factor, the subscript 0 indicates the initial parameter value, *t* is the blanching time, and *k* indicates the speed constant at the temperature used. The subscript eq indicates the equilibrium value. The effect of temperature on the rate constants *k* was described by Arrhenius’ law Equation (8), where *ke* is the pre-exponential constant; *Ea* is the activation energy (obtained through linear regression, estimated directly from the experimental data in one step—quality factor versus time, at all temperatures); *R* is the universal gas constant (8.3145 J mol^−1^ K^−1^); and *T* is the absolute temperature (in kelvin) [12].

(8) $$k=ke\left[-\frac{E_{a}}{R}\;\left(\frac{1}{T}\right)\right]$$

### 2.3. Methods Phase 2—Determining the Shelf-Life of the Product Stored under Refrigeration

#### 2.3.1. Determination of the Product’s Shelf-Life Stored under Refrigeration

The samples used to determine the product’s shelf-life stored under refrigeration: in natura (fresh) cowpeas (FC)—control, and blanched fresh cowpeas (BFC). The temperature–time binomial used for blanching met the following criteria: significant reduction in enzyme activity; minor change in firmness; moderate mass gain; and color indices with similar parameters to the control sample or with higher L*, more negative a*, more positive b*, and chroma with positive values. The FC and BFC samples were randomly packed in polyethylene plastic bags with 200 g each and stored randomly for a period of 14 days under refrigeration at 4.90 °C (0.32).

Physical–chemical and microbiological analyses, determination of enzymatic activity and photographic monitoring were performed every two days (0, 2, 4, 6, 8, 10, 12, and 14) in order to assess the changes tolerated by the product and to determine the shelf-life of FC and BFC. The samples were randomly selected each day for analysis by drawing lots with a completely randomized design.

#### 2.3.2. Physical–Chemical Analysis

The pH analysis was performed using a bench pH meter (pH21-01, Hanna Instruments^®^, São Paulo, Brazil) with a glass electrode, calibrated with buffer solutions (4 and 7). Titratable acidity was determined by volumetry using phenolphthalein as an indicator and the result was expressed in g of citric acid percentage [20].

#### 2.3.3. Microbiological Analysis

Total mesophilic and psychrotrophic aerobic counts were performed, the most likely number (MLN) was determined for coliforms at 45 °C and *Escherichia coli* (*E. coli*), coagulase positive staphylococci count/g [21] and *Salmonella* sp./25 g [22].

#### 2.3.4. Photographic Monitoring of Samples Stored under Refrigeration

A photographic record was performed to monitor changes in senescence during refrigerated storage, such as: color degradation, shedding of the integument, wounds, wrinkling, rot and foam formation. The samples (100 g) were put in a Petri dish placed in the center of a portable photographic studio (Pop Up Studio 35, Mutu^®^, São Paulo, Brazil) using a white background and standard LED light with a power of 5600 Lumens. Three records of each sample were taken with the objective of obtaining the one which best suited the technical requirements of image quality (resolution, standard lighting adjustment, and photo angle).

### 2.4. Statistical Analysis

The assays were performed randomly in duplicate and the samples were analyzed in triplicate. The assumptions for parametric analyzes were tested using the Shapiro–Wilk test. Fixed-effect ANOVA was performed considering a 95% confidence interval to assess the effects of the independent variables on the response variables. Tukey’s test was used to assess differences in means. The Student’s *t*-test was used to compare the means of treatments with the control group. A significance level of 5% (*p* < 0.05) was adopted for all analyses. The data were statistically analyzed using the Action Stat software program (Action Stat 3.0, Estatcamp^®^, São Paulo, Brazil).

## 3. Results and Discussion

### 3.1. Phase 1—Studying the Effects of Blanching on Fresh Cowpeas and a Kinetic Evaluation of the Variables Enzyme Activity, Firmness and Color

#### 3.1.1. Blanching Effect on Peroxidase Activity

**Figure 1:**
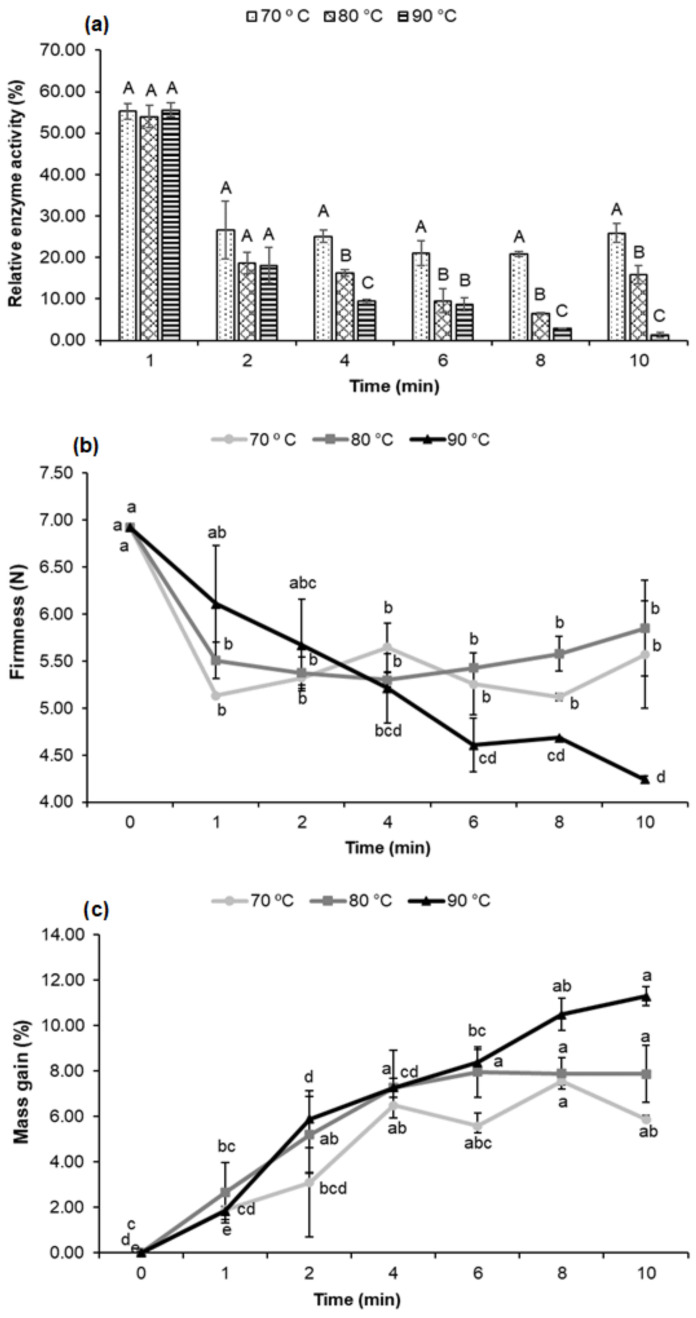
Effect of temperature and blanching time on the relative enzymatic activity (%) of peroxidase (**a**), firmness (N) (**b**) and on the mass gain (%) of fresh cowpeas (**c**). Notes: Mean of two samples and standard deviation bar. REA (%) and Mass gain (%): Each sample was analyzed in triplicate. Firmness (N): Each sample was composed of 15 cowpeas. ^ABC^ Means with different letters in each blanching time (min) differ significantly (*p* < 0.05) according to the Tukey test. ^abcd^ Means with different letters in the same line differ significantly (*p* < 0.05) according to the Tukey test.

An REA of 55.24% was observed in samples submitted to 70 °C for 1 min, with no significant difference (*p* > 0.05) when compared to 80 and 90 °C (Figure 1a). However, 80 and 90 °C temperatures revealed significant POD inhibition (*p* < 0.05) after 4 min of treatment in relation to 70 °C (Figure 1a). The lowest POD REA occurred with treatments of 90 °C for 8 min (2.94%) and for 10 min (1.25%) (Table S1).

The results were similar to those obtained in the study by Ruiz-Ojeda and Penãs [23], in which they found that the temperature of fresh cowpea pods must be around 90 °C to inhibit POD activity. Another study with green beans reported that 90% of the peroxidase inactivation was achieved by a blanching treatment at 90 °C for 3 min, but there was degradation in the chlorophyll contents in this binomial when compared to the control sample (in natura legume) [24].

The denaturation of the enzyme with the increase in temperature can be elucidated by the biphasic character of isoenzymes. Most of the heat-labile fractions are inactivated in the initial phase, while only the heat-resistant fractions remain active in the final stage and requires an increase in exposure time and an increase in temperature to completely inactivate or decrease the REA as much as possible [25].

Several studies have reported that the best choice for food blanching is based on enzymatic inactivation, or a reduction of at least 90% of peroxidase activity is recommended [11,14,26]. However, it is important to remember that quality aspects such as color and texture will directly influence the choice of the best pre-treatment [15,27].

#### 3.1.2. Blanching Effect on the Firmness

The temperatures of 70 and 80 °C caused less firmness loss; however, the increase in time and temperature to 90 °C more significantly influenced the decrease in firmness (Figure 1b and Table S2).

The decrease in firmness is due to the pectic polymers of the cell wall and middle lamella which change during processing [28]. Methoxylated pectin is predisposed to non-enzymatic depolymerization and demethoxylation conversions at high temperatures, mainly responsible for extensive softening of fresh foods. Furthermore, β depolymerization is affected in foods with low acid content during heat treatment [29].

Blanching can destroy the cell structure causing the vegetable to soften, so the best temperature–time binomial should be investigated. Research with fresh green peas found that the blanching temperature had a significant effect (*p* < 0.05) on the hardness, adhesiveness, and cohesiveness of the samples when heat treatment above 75 °C was applied [13].

Firmness is a fundamental quality attribute in the agri-food chain and its maintenance is important for ensuring food with desirable characteristics.

#### 3.1.3. Blanching Effect on Mass Gain

The lowest mass gain was at 70 °C (Figure 1c). There was a significant increase (*p* < 0.05) at 80 °C of up to 4 min of blanching and remained unchanged at other times. The greatest mass gain occurred between the temperatures studied at 90 °C, and the data show a significant increase (*p* < 0.05) in mass gain with the increase in blanching time.

This effect is justified by the application of moist heat during the heat treatment which gives greater yield from hydrating the fresh cowpeas, probably due to the filling of capillaries on the surface of the husks and in the grain hilum [30]. The greater mass gain at elevated temperatures is due to the increase in the molecular diffusion rate, increasing the water absorption capacity [31].

Joshi et al. [32] in researching water absorption and its impact on the texture of lentils (*Lens culinaris*), observed that French-green legumes absorbed the greatest amount of water when the immersion temperature was raised from 50 °C to 85 °C, attributing the effect to increased water permeability of the seed coat, seed surface area, high protein content, and relatively thinner seed coat.

In the present study, the maximum mass gain of 11.30% (90 °C for 10 min) is in the range of 10 to 15% that is considered acceptable for profitability of the process from an industrial point of view [23]. However, other quality factors must be considered for immature cowpeas. It was shown that the greater the mass gain, the lower the fresh cowpea firmness (r = −0.64 and *p* < 0.0001), as the swelling caused by water absorption consequently decreases the firmness (Table S3).

#### 3.1.4. Blanching Effect on Fresh Cowpea Color Properties

**Figure 2:**
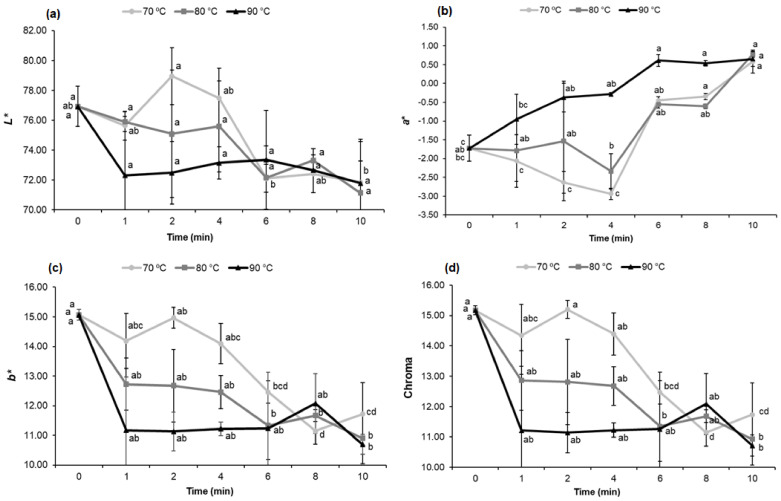
Effect of temperature and blanching time on color indexes (**a**) L*, (**b**) a*, (**c**) b* and (**d**) chroma of fresh cowpeas. Notes: Mean of two samples and standard deviation bar. Each sample was analyzed in triplicate. ^abcd^ Means with different letters in the same line differ significantly (*p* < 0.05) according to the Tukey test.

Fresh cowpeas become darker and opaque with an increase in temperature and blanching time, which corresponds to a decrease in the L* values, more positive a* values indicating a loss of green, and lower b* values, meaning with a more bluish hue, which causes fresh cowpeas to darken (Figure 2 and Table S4).

The best results were noticed in the 70 °C—2 min and 70 °C—4 min binomials, as they presented a more negative a*, meaning with a more accentuated gradation toward green (Figure 2b). In these binomials, the L* and a* values were better when compared to the control sample (FC), which made the blanched fresh cowpeas (BFC) have a better appearance and a more visible and brighter green tone.

The lower color intensity was noticed in extreme treatment conditions. This effect is evidenced in the chroma values, where less saturation is observed with an increase in temperature and time (Figure 2d).

This phenomenon probably occurred due to cell collapse and liquid exudation, inducing a lower reflectance of the sample [27].

The damage caused during the blanching can be due to the conversion of chlorophyll to pheophytin and pheoforbide, which results in a change from bright green to opaque olive green [33], as well as by the non-enzymatic action which involves phenolic compounds as substrates, the interaction of metal ions with phenolic compounds, and the Maillard reaction [34].

The best green color indexes were attributed to an expulsion of air trapped in the vegetable tissues around the food surface, which produces a change in the reflective properties of the vegetable [35].

Another possibility is that there is a gradual destruction of cell membranes from the appropriate heat treatment in fresh products, resulting in contact between enzymes and chlorophyll precursor compounds present in different organelles, and the general outcome is that the green color is accentuated during the first mild heat treatment periods [36].

Zhang et al. [13] stated that the increased green color perception in fresh peas after blanching was due to chlorophyll output as a result of thermal damage to the cell membrane and matrix structure, in addition the heat treatment expels gases between the plant tissues, condensing the green color.

Color is one of the main indicators used to assess the heat treatment intensity and to predict the degradation in quality caused by the blanching, because changes in this parameter can lead to decreased consumer acceptance, as well as causing commercial devaluation [9].

#### 3.1.5. Study of Kinetic Parameters in the Peroxidase (POD) Enzymatic Activity of Fresh Cowpeas

**Figure 3:**
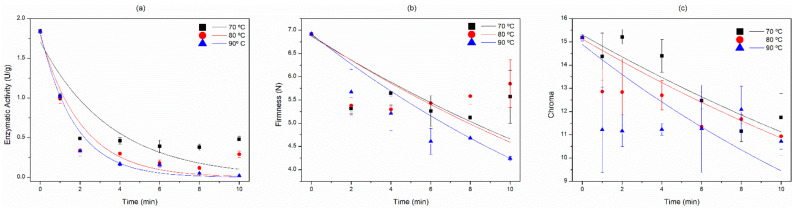
Effect of blanching fresh cowpeas in different temperature–time binomials under denaturation on: (**a**) enzymatic activity (U/g), (**b**) firmness (N), and (**c**) color saturation according to the chroma indicator.

The experimental results of the fresh cowpea peroxidase enzymatic activity after the blanching adjusted to the first order kinetic model with a high coefficient of determination (*R*^2^) at all studied temperatures and presenting values from 0.856 to 0.990 (Table 1 and Figure 3a). Thus, it is possible to confirm that there is temperature dependence for the enzyme denaturation which has a strong correlation.

The increase in blanching temperature led to an increase in kinetic constant k, varying from 0.281 to 0.581 at temperatures of 70, 80, and 90 °C, showing a more pronounced decrease in POD activity when the FC were subjected to the highest temperature (Table 1).

The estimated activation energy to decrease peroxidase activity was 36.95 kJ/mol. Thus far, no Ea results for FC have been recognized in the specialized literature.

#### 3.1.6. Kinetic Analysis of the Blanching Effect on Firmness

The decrease in firmness after the blanching in FC followed the first order kinetic reaction. The good quality of the data fit to the model can be seen by the *R*^2^ values and the behavior of k (Table 1).

The dependence of the velocity constant (*k*) with the temperature for the firmness has been well described by the Arrhenius equation. The obtained values reveal that the 70 °C temperature had less effect on deteriorating the firmness, followed by the 80 °C temperature with an intermediate reaction speed, and at 90 °C with the highest reaction speed to decrease the firmness of the FC (Table 1). Thus, it is observed that this quality attribute degraded with the heat treatment temperature increase (Figure 3b).

The resulting activation energy for BFC in the firmness property was 11.60 kJ/mol. This shows that this quality characteristic of fresh cowpeas is highly sensitive to heat, favoring application of mild temperatures in order to less deteriorate firmness.

#### 3.1.7. Kinetic Analysis of the Blanching Effect on Color

The experimental data adjusted satisfactorily to the first order kinetic model. The coefficient of determination (*R*^2^) values confirm the fit to the model (Table 1).

The *k* constant increases from 0.032 to 0.045 min^−1^ with an increase in temperature from 70 to 90 °C, showing that there is a dependence on the reaction speed with the temperature, generating fresh cowpeas with a dark hue when high temperatures and/or excessive processing time were used. Thus, the kinetic study demonstrated that the use of mild temperature and time favors maintaining intensity and better appearance of the studied sample. Blanching at 70 °C for 2 and 4 min showed the highest chroma values (Figure 3c), indicating color saturation, and this effect was confirmed by *k*.

The higher the chroma indicator values, the more desirable the food product is, as it shows a greater intensity and color saturation [12].

The activation energy was calculated by the natural logarithm linear regression of the kinetic constant *k* as a function of the inverse of the blanching temperature, which resulted in the value of 17.12 kJ/mol. The estimated *E_a_* indicates that the variation in color properties based on the chroma index is sensitive to the blanching temperature.

The symbols correspond to the mean data of two samples. Each sample was analyzed in triplicate. Firmness (N): Each sample was composed of 15 fresh cowpeas. The lines are the first order kinetic models.

#### 3.1.8. Defining the Ideal Blanching Conditions

The ideal blanching conditions were observed in the treatment at 70 °C for 4 min. This experimental binomial showed an REA% of 25.13, provided less decrease in firmness, moderate mass gain without compromising the consistency of the raw material, more saturated color, and with the most negative a*, indicating an intensification of the green tint in the fresh cowpeas. Thus, this blanching condition was adopted to determine the shelf-life of the product stored under refrigeration.

### 3.2. Phase 2—Determining the Shelf-Life of the Product Stored under Refrigeration

#### 3.2.1. Physical–Chemical Analysis of Fresh Cowpeas during Refrigeration Storage

**Figure 4:**
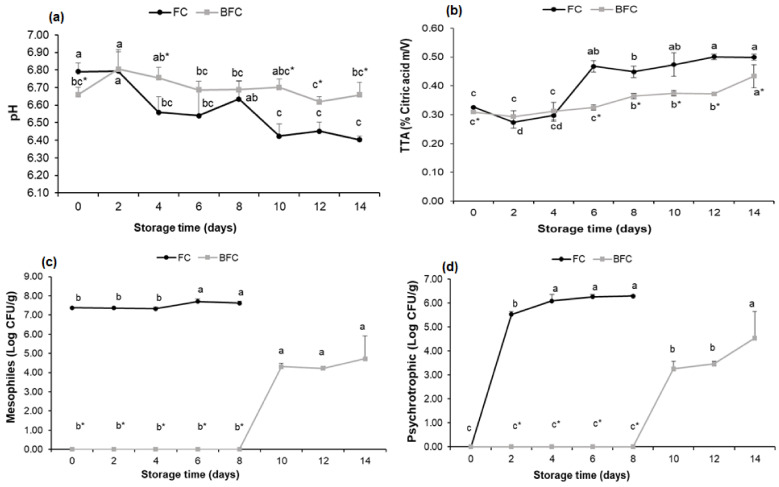
Effect of refrigerated storage (4.90 °C) on: (**a**) hydrogen potential (pH), (**b**) total titratable acidity (TTA), (**c**) on the mesophiles (Log CFU/g) and (**d**) psychrotrophic bacteria (Log CFU/g) counts of fresh cowpea (FC) and blanched fresh cowpea (BFC) samples at 70 °C for 4 min. Mean of two samples and standard deviation bar. Each sample was analyzed in triplicate. ^abcd^ Means with different letters in the same line differ significantly (*p* < 0.05) according to the Tukey test. * Means followed by an asterisk differ significantly (*p* < 0.05) from the control (FC) according to the Student’s *t*-test.

The FC showed greater pH instability during the refrigerated storage period, with a significant decrease (*p* < 0.05) after 4 days of storage, reaching pH stability from day 10, but with a considerable decrease when compared to the initial value. This decrease in the pH of the control sample shows acidification of the FC, probably due to the senescence process and/or microbial growth (Figure 4a and Table S5).

The pH of the BFC significantly differed (*p* < 0.05) from the control sample on day 0, with a lower value (Figure 4a and Table S5). However, it showed a significant increase (*p* < 0.05) after 2 days of storage which remained without major changes until the end of the storage period, being significantly (*p* < 0.05) higher than the pH of FC at times 4, 10, 12, and 14. Thus, the raw material is classified as a food with low acidity due to the presented pH values.

In the total titratable acidity (TTA) analysis, it was found that the FC and BFC samples exhibited low acidity levels, corroborating with the pH results (Figure 4b and Table S5). The FC showed a significant increase (*p* < 0.05) in the TTA from day 6, being equivalent to the decrease in the pH observed for the same period. The acidity levels for the BFC sample did not show significant differences (*p* > 0.05) until the sixth day of analysis, with a variation on day 8, then remaining stable until day 12, and differing significantly (*p* < 0.05) in the final 14 days of storage. However, the BFC sample differed significantly (*p* < 0.05) from the control (FC) from day 6 onwards, once again evidencing stability of the TTA in the BFC and acidification of the FC when compared to the BFC.

#### 3.2.2. Microbiological Quality during Refrigeration Storage

The in natura cowpeas and the blanched sample presented satisfactory results and acceptable quality during the whole storage period (absence of *Salmonella* in 25 g, *E. coli* count < 3.0 MLN/g and absence of coagulase Staphylococci positive/g). These results indicate that there was no post-process contamination.

However, there were high counts of the coliforms at 45 °C (>1100 MLN/g) in FC samples from day 2 (Table S6) until the last day of storage, indicating the recovery capacity of injured cells after a short period of time, and this contamination probably occurred due to inadequate hygiene conditions during the threshing and/or bagging process of fresh cowpeas shortly after harvest. The temperature–time binomial (70 °C—4 min) applied in the fresh cowpea blanching proved to be effective for inactivating this group of microorganisms and was a favorable indicator of the hygiene conditions of the pretreatment heating process. Coliforms are easily destroyed by heat and must not survive the heat treatment and consequently the storage period, as the presence of these microorganisms can be indicative of post-process contamination [37].

The control sample (FC) showed a high microbial load of mesophilic aerobes from the initial period (day 0) with 7.38 Log CFU/g, and the multiplication increased significantly (*p* < 0.05) from day 6 until the end of the storage period. The BFC significantly differed (*p* < 0.05) from the FC, since the blanching reduced the microbial load. The BFC sample showed a significant count (*p* < 0.05) of 4.30 Log CFU/g on the 10th day of refrigerated storage, and there was no significant difference (*p* > 0.05) until the 14th day of refrigerated storage (Figure 4c).

The FC sample did not show a count of psychrotrophic microorganisms on day 0. However, a significant logarithmic multiplication (*p* < 0.05) with 5.53 Log CFU/g was observed starting from the second day of storage, and by day 4 there was a significant count (*p* < 0.05) of 6.09 Log CFU/g, with no significant difference (*p* > 0.05) until the 8th day of cold storage (Figure 4d).

The blanching decreased the microbiological load of the BFC sample, and maintained stability until day 8, with no significant difference (*p* > 0.05). A significant multiplication (*p* < 0.05) was observed on day 10 of refrigerated storage, maintaining a high count until the last day of storage.

LaFountain et al. [38] investigated improved safety and shelf-life of chilled cucumber pickles, and found that blanching in water at 80 °C for 90 s achieved a minimum 2 log reduction in cucumber microbiota and a 5 log reduction in *Escherichia coli* O157:H7.

It is possible to show that the reduction in the microbiological load after blanching application delayed the microbiological multiplication, proving to be effective in preventing deterioration of the fresh cowpeas until the 8th day of refrigerated storage. Although the legume needs effective heat treatment prior to consumption, the use of food with appropriate microbiological standards is essential to ensuring food safety, as well as to maintaining the shelf-life of the raw material.

#### 3.2.3. Change in Peroxidase Activity during the Fresh Cowpea Cold Storage Period

FC peroxidase activity showed a significant decrease (*p* < 0.05) from day 10 of refrigerated storage when compared to the initial period (Table 2).

The effect in decreasing POD activity may be correlated with the decrease in the polyphenol levels during the senescence process, since the degradation of phenolic compounds can bind with other cellular components and make it unavailable to the POD enzyme action [39].

Fresh cowpea blanching at 70 °C for 4 min provided an effective reduction in POD activity and stability until the 8th day, with no significant difference (*p* > 0.05). However, a significant increase (*p* < 0.05) of peroxidase activity was observed from the 10th day of refrigerated storage (Table 2).

Considering that the thermal inactivation of the enzymes is reversible, mainly of POD [24], it is important to know the ascendancy time of this activity in order to establish quality standards and define the shelf-life of the raw material.

The POD effects heated in an aqueous medium alters the stabilization of interactions within the molecule and the enzyme configuration, causing inactivation, but the molecules can recover their activity when the solution cools, as partially inactivated isoenzymes can recover activity depending on time and storage temperature [40].

An interesting finding in the present study sparked an investigation of other hypotheses which may contribute to increasing this enzymatic activity. It is valid to ponder microbiological observations and correlate them with POD activity. The BFC showed a significant multiplication (*p* < 0.05) of mesophilic and psychrotrophic bacteria from the 10th day of analysis, also corresponding to the ascendency of POD.

The protective function of some oxidative peroxidases has been reported as a defense system in plants [41], because POD catalyzes peroxide reduction and generates reactive oxygen species when infested [42].

In this context, the increase in enzyme activity observed after the 10th day of refrigeration for the BFC sample may have been induced by the renaturation, configuration, and thermostability of the isoenzymes; by the increase in microbial count; or even by the association of factors.

#### 3.2.4. Photographic Monitoring

**Figure 5:**
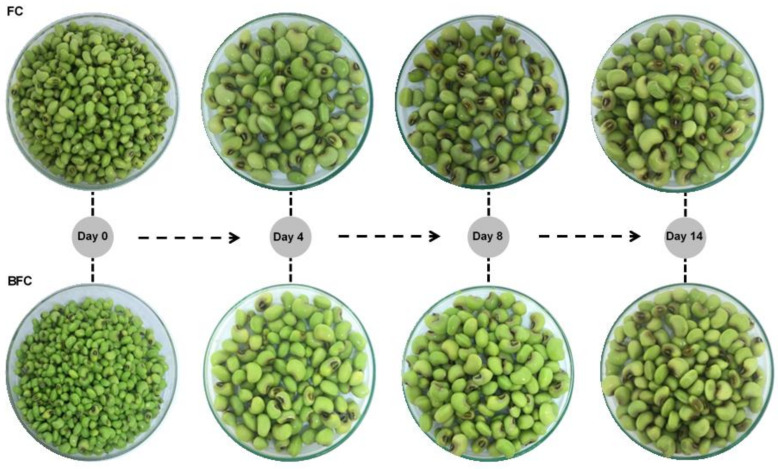
Photographic monitoring of senescence stage of fresh cowpeas (FC) and blanched fresh cowpeas (BFC) stored under refrigeration.

The control sample (FC) started the degradation processes from day 4 of storage with a compromise in the fresh cowpea structure, such as: shedding the integument and small oxidation in the halo, causing darkening. From the 8th day onwards, it was also possible to observe the appearance of wounds, foam formation, and changes in color, with more yellow grains due to the decrease in the chlorophyll pigment. The last day (14) presented rotting grains with more severe oxidation and darkening processes, wrinkling, softening, and viscous liquid (Figure 5).

These deterioration effects can be related to several factors because the quality attributes (color, firmness, and flavor) in untreated vegetables after harvesting can be affected by the action of intracellular enzymes, such as peroxidase and polyphenoloxidase, by the action of microorganisms and by physical–chemical changes [9].

The blanched sample (BFC) exhibited greater color uniformity throughout the storage period (Figure 5). Deterioration characteristics were observed in the last three days of storage, showing darkening of the halo, slight wrinkling, and liquid exudation. Thus, we can consider that the blanching provided prolonged quality of the fresh cowpeas in the refrigerated storage period.

## 4. Conclusions

Blanching significantly decreased the peroxidase enzymatic activity, with the reduction being accentuated with the increase in the binomial temperature and time. The first order kinetic modeling enabled inferring that the blanching binomial at 70 °C for 4 min was adequate to stabilize the enzymatic deterioration, also minimizing important losses in quality such as moderate mass gain and less firmness loss, and improved the green color intensity of the fresh cowpeas.

The blanching treatment logarithmically reduced the microbial load of fresh cowpeas and the variations in the physicochemical analysis of pH and total titratable acidity were minimal during the storage period. The blanched sample (BFC) ensured the quality of the studied parameters until the 8th day of storage under refrigeration at 4.90 °C (0.32), extending the shelf-life of the samples by 5 days. Blanching application is an alternative for improving fresh cowpeas, better guaranteeing quality and safety attributes, as well as minimizing waste due to high perishability of fresh grains.

## Figures and Tables

**Table 1:** First order kinetic parameters for estimating peroxidase denaturation (U/g), firmness property (N), and color change (chroma index) of fresh cowpeas submitted to blanching at different temperatures.

Variables and Blanching Temperature (°C)	*C*_0_	*k* (min^−1^)	*R* ^2^	*E_a_* (kJ/mol)
**Peroxidase (U/g)**				
70	1.71 (0.18)	0.281 (0.084)	0.856	36.95
80	1.80 (0.15)	0.484 (0.082)	0.952	
90	1.83 (0.08)	0.581 (0.057)	0.990	
**Firmness (N)**				
70	6.87 (0.13)	3.89 × 10^−2^ (0.70 × 10^−2^)	0.903	
80	6.90 (0.11)	4.07 × 10^−2^ (0.42 × 10^−2^)	0.960	11.60
90	6.92 (0.03)	4.89 × 10^−2^ (0.058 × 10^−2^)	0.999	
**Color (chroma)**				
70	15.30 (0.22)	0.032 (0.005)	0.890	
80	15.13 (0.15)	0.033 (0.003)	0.974	17.12
90	14.89 (0.47)	0.045 (0.011)	0.806	

Mean of two samples. Each sample was analyzed in triplicate. The values expressed are means (standard error). *C*_0_—initial value of the quality factor; *k* (min^−1^)—reaction speed constant at the temperature used. *R*^2^—coefficient of determination. *E_a_* (kJ/mol)—activation energy.

**Table 2:** Effect of blanching on the peroxidase enzymatic activity (U/g) during the refrigeration storage period of fresh cowpeas (FC) and blanched fresh cowpeas (BFC).

Variable and Samples			Storage Time (Days)					
	0	2	4	6	8	10	12	14
**Peroxidase** **activity (U/g)**								
**FC**	3.15 ^a^ (0.40)	3.10 ^a^ (0.35)	2.95 ^ab^ (0.45)	2.51 ^abc^ (0.45)	2.59 ^abc^ (0.38)	2.06 ^c^ (0.61)	2.12 ^c^ (0.14)	2.24 ^bc^ (0.07)
**BFC**	1.85 ^d^* (0.04)	1.82 ^d^* (0.07)	1.85 ^d^* (0.03)	1.84 ^d^* (0.52)	1.97 ^cd^* (0.44)	3.15 ^ab^* (0.22)	2.58 ^bc^ (0.56)	3.62 ^a^* (0.31)

Mean of two samples (standard deviation). Each sample was analyzed in triplicate. ^abcd^ Means with different letters in the same line differ significantly (*p* < 0.05) according to the Tukey test. * Means followed by an asterisk differ significantly (*p* < 0.05) from the control sample (FC) according to the Student’s *t*-test.

## Data Availability

The most of data is contained within the Supplementary Materials.

## Supplementary Materials

The following supporting information can be downloaded at: https://www.mdpi.com/article/10.3390/foods11091295/s1. Figure S1: Position of fresh cowpeas for the instrumental firmness analysis. Adapted from [19]. Table S1: Effect of temperature and blanching time on the relative enzymatic activity (REA %) of fresh cowpea peroxidase. Table S2: Effect of temperature and blanching time on the firmness property (N) and mass gain (%) of fresh cowpeas. Table S3: Pearson’s correlation between the mass gain parameter and firmness of fresh cowpeas submitted to blanching. Table S4: Effect of temperature and blanching time on fresh cowpea color according to the *L**, *a**, *b** and chroma indices. Table S5: Effect of refrigerated storage (4.90 °C) on hydrogen potential (pH), total titratable acidity (TTA), mesophilic (Log CFU/g) and psychrotrophic (Log CFU/g) bacteria in the fresh cowpea (FC) and blanched fresh cowpea (BFC) samples at 70 °C for 4 min. Table S6: Coliform count at 45 °C during the refrigerated storage period of fresh cowpeas (FC) and blanched fresh cowpeas (BFC).
