# Supplementary material for: Blanching Effect on the Quality and Shelf-Life Characteristics of Fresh Cowpea Grains [Vigna unguiculata (L.) Walp.]
Source: Foods. 2022 Apr 29;11(9):1295. doi: 10.3390/foods11091295 (PMC9100185; doi:10.3390/foods11091295)
Supplement: Supplementary file 1 [file foods-11-01295-s001.zip › Supplementary Figure e Tables Legends.pdf]

Supplementary Figure Legend

Figure S1. Position of fresh cowpeas for the instrumental firmness analysis.

P1 - length, smallest axis; P2 - width, middle axis; P3 - height, longest axis
Source: Adapted from [19]

Supplementary Tables Legends

Table S1. Effect of temperature and blanching time on the relative enzymatic activity (REA %) of fresh cowpea peroxidase.

Means of two samples (standard deviation). Each sample was analyzed in triplicate.

$^{abc.}$ Means with different letters in the same line differ significantly ($p<0.05$) according to the Tukey test.

$^{ABC.}$ Means with different capital letters in the same column for each variable differ significantly ($p<0.05$) according to the Tukey test.

Table S2. Effect of temperature and blanching time on the firmness property (N) and mass gain (%) of fresh cowpeas.

Means of two samples (standard deviation). Mass gain (%): Each sample was analyzed in triplicate. Firmness (N): Each sample was composed of 15 cowpeas.

$^{abc.}$ Means with different letters in the same line differ significantly ($p<0.05$) according to the Tukey test.

$^{ABC.}$ Means with different capital letters in the same column for each variable differ significantly ($p<0.05$) according to the Tukey test.

Table S3. Pearson's correlation between the mass gain parameter and firmness of fresh cowpeas submitted to blanching.

Table S4. Effect of temperature and blanching time on fresh cowpea color according to the L^*, a^*, b^* and chroma indices.

Means of two samples (standard deviation). Each sample was analyzed in triplicate.

$^{abc.}$ Means with different letters in the same line differ significantly ($p<0.05$) according to the Tukey test.

$^{ABC.}$ Means with different capital letters in the same column for each variable differ significantly ($p<0.05$) according to the Tukey test.

Table S5. Effect of refrigerated storage (4.90 °C) on hydrogen potential (pH), Total Titratable Acidity (TTA), mesophilic (Log CFU/g) and psychrotrophic (Log CFU/g) bacteria in the fresh cowpea (FC) and blanched fresh cowpea (BFC) samples at 70 °C for 4 min.

Means of two samples (standard deviation). Each sample was analyzed in triplicate.

$^{abc.}$ Means with different letters in the same line differ significantly ($p<0.05$) according to the Tukey test.

*Means followed by an asterisk differ significantly ($p<0.05$) from the control sample (FC) according to the Student's t-test.

Table S6. Coliform count at 45 °C during the refrigerated storage period of fresh cowpeas (FC) and blanched fresh cowpeas (BFC).

Means of two samples. Each sample was analyzed in triplicate.
