# Supplementary material for: Blanching Effect on the Quality and Shelf-Life Characteristics of Fresh Cowpea Grains [Vigna unguiculata (L.) Walp.]
Source: Foods. 2022 Apr 29;11(9):1295. doi: 10.3390/foods11091295 (PMC9100185; doi:10.3390/foods11091295)
Supplement: Supplementary file 1 [file foods-11-01295-s001.zip › Table S1.pdf]

Table S1. Effect of temperature and blanching time on the relative enzymatic activity (REA %) of fresh cowpeas peroxidase.

Variables and Blanching Temperature (°C)	Blanching time (minutes)						
	0	1	2	4	6	8	10
**REA (%)**							
70	100^{a}	55.24^{bA} (1.85)	26.56^{cA} (6.99)	25.13^{cA} (1.55)	21.00^{cA} (2.99)	20.88^{cA} (0.60)	25.87^{cA} (2.33)
80	100^{a}	53.99^{bA} (2.64)	18.72^{cA} (2.61)	16.28^{cdB} (0.75)	9.55^{deB} (2.93)	6.49^{eB} (0.21)	15.83^{cdB} (2.24)
90	100^{a}	55.58^{bA} (1.67)	18.10^{cA} (4.30)	9.40^{dC} (0.50)	8.71^{dB} (1.61)	2.94^{deC} (0.08)	1.25^{eC} (0.58)

Means of two samples (standard deviation). Each sample was analyzed in triplicate.

$^{abc.}$ Means with different letters in the same line differ significantly (p<0.05) according to the Tukey test.

$^{ABC.}$ Means with different capital letters in the same column for each variable differ significantly (p<0.05) according to the Tukey test.
