# Supplementary material for: Blanching Effect on the Quality and Shelf-Life Characteristics of Fresh Cowpea Grains [Vigna unguiculata (L.) Walp.]
Source: Foods. 2022 Apr 29;11(9):1295. doi: 10.3390/foods11091295 (PMC9100185; doi:10.3390/foods11091295)
Supplement: Supplementary file 1 [file foods-11-01295-s001.zip › Table S2.pdf]

Table S2. Effect of temperature and blanching time on the firmness property (N) and mass gain (%) of fresh cowpeas.

Variables and Blanching Temperature (°C)	Blanching time (minutes)						
	0	1	2	4	6	8	10
**Firmness (N)**							
70	6.92^{a} (0.02)	5.13^{bA} (0.01)	5.32^{bA} (0.08)	5.65^{bA} (0.26)	5.26^{bA} (0.33)	5.12^{bB} (0.04)	5.57^{bA} (0.57)
80	6.92^{a} (0.02)	5.51^{bA} (0.19)	5.38^{bA} (0.17)	5.30^{bA} (0.07)	5.43^{bA} (0.02)	5.58^{bA} (0.18)	5.85^{bA} (0.51)
90	6.92^{a} (0.02)	6.11^{abA} (0.62)	5.67^{abcA} (0.49)	5.21^{bcdA} (0.37)	4.61^{cdA} (0.28)	4.68^{cdB} (0.01)	4.24^{dA} (0.04)
**Mass gain (%)**							
70	0.00^{d}	1.89^{cdA} (0.15)	3.10^{bcdA} (0.43)	6.50^{abA} (2.40)	5.58^{abcA} (0.56)	7.56^{aB} (0.29)	5.88^{abB} (0.15)
80	0.00^{c}	2.66^{bcA} (1.33)	5.20^{abA} (1.70)	7.27^{aA} (0.42)	7.96^{aA} (1.11)	7.89^{aB} (0.70)	7.88^{aB} (1.25)
90	0.00^{e}	1.85^{eA} (0.07)	5.89^{dA} (1.27)	7.29^{cdA} (0.14)	8.39^{bcA} (0.58)	10.50^{adA} (0.71)	11.30^{aA} (0.42)

Means of two samples (standard deviation). Mass gain (%): Each sample was analyzed in triplicate. Firmness (N): Each sample was composed of 15 cowpeas.
$^{abc.}$ Means with different letters in the same line differ significantly ($p<0.05$) according to the Tukey test.
$^{ABC.}$ Means with different capital letters in the same column for each variable differ significantly ($p<0.05$) according to the Tukey test.
