# Supplementary material for: Blanching Effect on the Quality and Shelf-Life Characteristics of Fresh Cowpea Grains [Vigna unguiculata (L.) Walp.]
Source: Foods. 2022 Apr 29;11(9):1295. doi: 10.3390/foods11091295 (PMC9100185; doi:10.3390/foods11091295)
Supplement: Supplementary file 1 [file foods-11-01295-s001.zip › Table S3.pdf]

Table S3. Pearson's correlation between the mass gain parameter and firmness of fresh cowpeas submitted to blanching.

**Variables**	**Mass Gain (%)**		**Firmness (N)**	
	**r**	**p-valor**	**r**	**p-valor**
**Mass gain (%)**	1	0	-0.640	< 0.0001
**Firmness (N)**	-0.640	< 0.0001	1	0
