# Supplementary material for: Blanching Effect on the Quality and Shelf-Life Characteristics of Fresh Cowpea Grains [Vigna unguiculata (L.) Walp.]
Source: Foods. 2022 Apr 29;11(9):1295. doi: 10.3390/foods11091295 (PMC9100185; doi:10.3390/foods11091295)
Supplement: Supplementary file 1 [file foods-11-01295-s001.zip › Table S4.pdf]

Table S4. Effect of temperature and blanching time on fresh cowpeas color according to the *L**, *a**, *b** and chroma indices.

Variables and Blanching Temperature (°C)	Blanching time (minutes)						
	**CONTROL**	**1**	**2**	**4**	**6**	**8**	**10**
L*							
70	76.93^{ab} (1.35)	75.63^{abA} (0.96)	78.95^{aA} (1.90)	77.47^{abA} (2.02)	72.10^{bA} (0.93)	72.40^{abA} (1.27)	71.85^{bA} (2.72)
80	76.93^{a} (1.35)	75.90^{aA} (0.67)	75.08^{aA} (4.27)	75.59^{aA} (3.05)	72.14^{aA} (2.14)	73.31^{aA} (0.77)	71.14^{aA} (2.14)
90	76.93^{a} (1.35)	72.30^{aA} (3.94)	72.47^{aA} (2.11)	73.15^{aA} (1.08)	73.36^{aA} (3.32)	72.66^{aA} (0.02)	71.79^{aA} (2.93)
a*							
70	-1.73^{b} (0.35)	-2.07^{cA} (0.70)	-2.63^{cA} (0.29)	-2.94^{cB} (0.15)	-0.46^{abB} (0.10)	-0.35^{aB} (0.09)	0.58^{aA} (0.31)
80	-1.73^{ab} (0.35)	-1.79^{abA} (0.82)	-1.54^{abA} (1.59)	-2.34^{bB} (0.48)	-0.55^{abB} (0.11)	-0.61^{abB} (0.07)	0.77^{aA} (0.07)
90	-1.73^{c} (0.35)	-0.95^{bcA} (0.67)	-0.38^{abA} (0.38)	-0.29^{abA} (0.04)	0.61^{aA} (0.16)	0.54^{aA} (0.07)	0.66^{aA} (0.21)
b*							
70	15.07^{a} (0.18)	14.19^{abcA} (0.93)	14.97^{abA} (0.35)	14.09^{abcA} (0.68)	12.46^{bcdA} (0.38)	11.15^{dA} (0.45)	11.72^{cdA} (1.05)
80	15.07^{a} (0.18)	12.73^{abA} (0.88)	12.68^{abAB} (1.22)	12.46^{abAB} (0.56)	11.34^{bA} (1.14)	11.67^{bA} (0.20)	10.90^{bA} (0.85)
90	15.07^{a} (0.18)	11.17^{abA} (1.80)	11.13^{abB} (0.66)	11.22^{abB} (0.24)	11.24^{abA} (1.89)	12.08^{abA} (1.01)	10.70^{bA} (0.33)
**Chroma**							
70	15.07^{a} (0.14)	14.34^{abcA} (1.02)	15.20^{aA} (0.30)	14.39^{abA} (0.69)	12.47^{bcdA} (0.38)	11.15^{dA} (0.45)	11.74^{cdA} (1.04)
80	15.07^{a} (0.14)	12.86^{abA} (0.99)	12.81^{abAB} (1.40)	12.68^{abAB} (0.63)	11.35^{bA} (1.15)	11.68^{abA} (0.21)	10.93^{bA} (0.85)
90	15.07^{a} (0.14)	11.22^{abA} (1.85)	11.14^{abB} (0.67)	11.22^{abB} (0.24)	11.26^{abA} (1.87)	12.09^{abA} (1.00)	10.72^{aA} (0.34)

Means of two samples (standard deviation). Each sample was analyzed in triplicate.

$^{abc.}$ Means with different letters in the same line differ significantly ($p<0.05$) according to the Tukey test.

$^{ABC.}$ Means with different capital letters in the same column for each variable differ significantly ($p<0.05$) according to the Tukey test.
