# Supplementary material for: Blanching Effect on the Quality and Shelf-Life Characteristics of Fresh Cowpea Grains [Vigna unguiculata (L.) Walp.]
Source: Foods. 2022 Apr 29;11(9):1295. doi: 10.3390/foods11091295 (PMC9100185; doi:10.3390/foods11091295)
Supplement: Supplementary file 1 [file foods-11-01295-s001.zip › Table S5.pdf]

Table S5. Effect of refrigerated storage (4.90 °C) on hydrogen potential (pH), Total Titratable Acidity (TTA), mesophilic (Log CFU/g) and psychrotrophic (Log CFU/g) bacteria in the fresh cowpeas (FC) and blanched fresh cowpeas (BFC) samples at 70 °C for 4 min.

**Variable and Samples**	**Storage time (days)**							
	**0**	**2**	**4**	**6**	**8**	**10**	**12**	**14**
**pH**								
FC	6.79^{a} (0.05)	6.79^{a} (0.11)	6.56^{bc} (0.09)	6.54^{bc} (0.16)	6.64^{ab} (0.10)	6.42^{c} (0.07)	6.45^{c} (0.05)	6.40^{c} (0.02)
BFC	6.66^{bc*} (0.04)	6.81^{a} (0.11)	6.76^{ab*} (0.06)	6.69^{bc} (0.05)	6.69^{bc} (0.05)	6.70^{abc*} (0.05)	6.62^{c*} (0.03)	6.66^{bc*} (0.07)
**TTA (% Citric acid m/V)**								
FC	0.33^{c} (0.00)	0.27^{d} (0.02)	0.30^{cd} (0.02)	0.47^{ab} (0.02)	0.45^{b} (0.02)	0.47^{ab} (0.04)	0.50^{a} (0.01)	0.50^{a} (0.01)
BFC	0.31^{c*} (0.00)	0.29^{c} (0.02)	0.31^{c} (0.03)	0.32^{c*} (0.01)	0.36^{b*} (0.01)	0.37^{b*} (0.01)	0.37^{b*} (0.00)	0.43^{a*} (0.04)

Means of two samples (standard deviation). Each sample was analyzed in triplicate.

$^{abc.}$ Means with different letters in the same line differ significantly ($p<0.05$) according to the Tukey test.

*Means followed by an asterisk differ significantly ($p<0.05$) from the control sample (FC) according to the Student's t-test.
