# Supplementary material for: Blanching Effect on the Quality and Shelf-Life Characteristics of Fresh Cowpea Grains [Vigna unguiculata (L.) Walp.]
Source: Foods. 2022 Apr 29;11(9):1295. doi: 10.3390/foods11091295 (PMC9100185; doi:10.3390/foods11091295)
Supplement: Supplementary file 1 [file foods-11-01295-s001.zip › Table S6.pdf]

Table S6. Coliform count at 45 °C during the refrigerated storage period of fresh cowpeas (FC) and blanched fresh cowpeas (BFC).

Samples	Storage time (days)							
	**0**	**2**	**4**	**6**	**8**	**10**	**12**	**14**
**FC**	<3.0	>1,100	>1,100	>1,100	>1,100	-	-	-
**BFC**	<3.0	<3.0	<3.0	<3.0	<3.0	<3.0	<3.0	<3.0

Means of two samples. Each sample was analyzed in triplicate.
