# Supplementary figures and images for: Blanching Effect on the Quality and Shelf-Life Characteristics of Fresh Cowpea Grains [Vigna unguiculata (L.) Walp.]
Source: Foods. 2022 Apr 29;11(9):1295. doi: 10.3390/foods11091295 (PMC9100185; doi:10.3390/foods11091295)

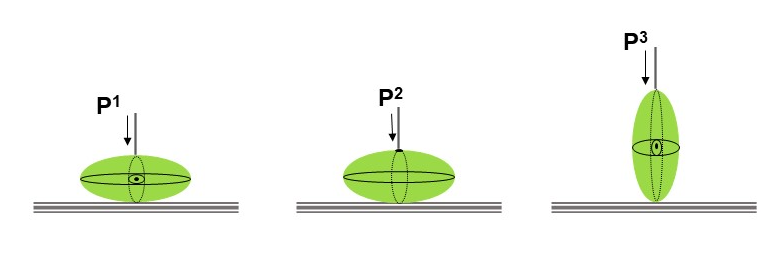

Supplement: Supplementary file 1 [file foods-11-01295-s001.zip › Supplementary Figure S1.tif]
